# Keystone Veterinary Medical Association

**Published:** 1893-01

**Authors:** W. S. Kooker

**Affiliations:** Secretary


W. S. KOOKER, *Secretary.*

---

*Keystone Veterinary Medical Association.*—The regular meeting of the Keystone Veterinary Medical Association was held December 19th, 1892, at the College of Physicians. The meeting was called to order by the President, Dr. R. G. Webster. The following answered roll call: Drs. Gentner, Hoskins, Raynor, Kooker, Bridge, Lintz, Sellers and Nunan. The application of Dr. Walter R. Hart and Dr. Robert J. Fox for membership was received, and that of Dr. John R. Hart for associate membership. They were referred to trustees to be reported at next meeting.

The Board of Trustees made the following report: "We, your Board of Trustees, after a thorough examination of the list of delinquents submitted to us by the Treasurer, and after having appealed to these delinquents by personal letter to each one, make the following recommendation: That the Secretary and Treasurer remove from their rolls the name of Dr. T. B. Rogers, whose resignation or expulsion is a matter of record ; the same disposition in regard to the name of Dr. W. H. Mattson, if the minutes of our Association show the acceptance of his resignation ; if said resignation was not accepted, owing to his initiation fee and dues not having been paid, we would therefore recommend that his name be included with the following for suspension for non-payment of initiation fees and dues until such arrearages are paid: Drs. R. W. Hickman, W. B. Montgomery, J. F. Vandergrift, Gulden R. Hartman, A. F. Schreiber, W. A. Birch, Antoni Maurice and E. H. Landis. Further, that Dr. J. T. Ferley be relieved of the assessment charged against him by our Treasurer, on the same grounds as Dr. Nunan. Further, that our association adopt a monthly system of dues, and that twenty-five cents be the sum decided upon. We also further recommend that at least one meeting of each year be devoted to a social reunion, in conjunction with an invitation to some prominent member of the profession to entertain us with a paper, to be followed by a banquet, and at this meeting to dispense entirely with the regular order of business after the calling of the roll. We further recommend that all members of the Association will endeavor to more promptly comply with the requirements of our Association in the payment of their dues, etc.

*Trustees,* { W. HORACE HOSKINS,

C. LINTZ,

J. B. RAYNOR,

A. T. SELLERS.

The following paper was then read by W. D. NUNAN:

### WOUNDS OF THE FEET—RESULTS AND TREATMENT.

As my time is limited I will endeavor to make the following article quite brief, with no attempt at elaboration. I believe a great mistake is frequently made by many veterinarians in the treatment of punctured wounds of the feet, by cutting into and exposing to the very bottom a foot after a simple puncture, for by so doing they necessitate frequent dressings, not to mention the possibility of serious complications. I know of cases where practitioners have laid open a foot, after a slight injury by a small nail or wire, when there was scarcely the slightest prospect of any foreign or deleterious matter contained therein, thus causing the animal to be laid up for a long time at considerable cost to the owner. Unless the object sought is to augment an otherwise nominal bill, this treatment is a mistake. Even at this it is an error, for such practices are to be avoided.

In regard to the use of the knife on the foot, if it were necessary I would cut down and lay it open as fearlessly as any other part of the economy.

I will cite a case I had during the past summer. About June 8th a gray mare belonging to Richardson Shoemaker, proprietor of the Lansdown Livery Stables, picked up a nail. It was a tenpenny nail, I believe, which entered near the apex of the frog, a little to the inside, passing up through the sole, the velvety tissue, and into the Os Pedis, as we afterward learned. After discovering the cause of the lameness, he endeavored to remove it and partially succeeded, inasmuch as after developments demonstrated that the nail broke and a portion remained imbedded in the pedal bone.

He took her to a blacksmith shop, and I suppose the blacksmith applied spirits of salts, etc., the usual remedies found in a shop.

The mare was driven about a week with variable degrees of lameness. Then the lameness became so severe, it being with the greatest difficulty that she could be induced to put the foot under her. On June 15th I was called to see her. I probed into the wound, and soon could discern the grating of two metals.

I then diagnosed that a portion of the nail had penetrated the Os Pedis and had become broken off. I cut down through the sole, the velvety tissue, and although there was considerable hæmorrhage, I continued cutting until I exposed the third phalanx.

I may mention incidentally that I would frequently attempt to grasp the nail with a small forceps. At last I succeeded and extracted it. The after treatment consisted of checking the hæmorrhage, opium, oil of tar, and a compress of cotton and oakum.

Giving the animal hyperæmic injections of hydrocyanic acid. I dressed the wound on the following day, then increased the interim between dressings. The exuberant granulations were considerable, completely filling up and overlapping the excised portion of the sole. The first few days I attempted to burn this down with Lunar Caustic, but I afterwards resorted to the actual cautery. I ordered the animal shod with leather under shoe, cotton and oil of tar dressing, and put to work, with instructions to remove shoe in the course of a week. The mare continued working, and traveled sound unless she would happen to stand on a loose stone.

Another error, and quite a common one with many, is poulticing a foot with flax-seed, etc. I don't mean to condemn the practice itself, but this is frequently overdone, with the result, instead of being beneficial, it is often conducive to the propagation of pus, and the hyper softening of the adjacent tissues not primarily injured, and ultimately a slough between hair and hoof, or some other untoward termination. Much better treatment is to pare down sufficiently at the orifice, to permit the free exit of all exudations (in slight traumations this sometimes may be dispensed with) and the introduction of nitric and carbolic acids.

Dr. Hoskins a short time since, at one of our meetings, recommended the application of the actual cautery. I believe the acid treatment will serve equally as well, with the great advantage of reaching to the very bottom and cauterizing all the tissue injured, and the theory now advanced of its being effectual against tetanic germs would also recommend it.

The results of punctured wounds of the feet are numerous. Invariably there is lameness if not treated. Frequently with even best treatment the horse will continue to limp, even after a slight puncture scarcely penetrating the sole. Some may wonder and become gravely apprehensive at this, but if they will only consult their anatomical knowledge they will readily appreciate the cause, being cognizant of the numerous nerve and blood supply, and as the traumation is invariably followed by inflammation with its well known characteristics, one of which, swelling causes pressure; and this highly nervous tissue being securely walled in by a horny box, the pressure is directly on the nerve filaments.

After an injury, if the animal is of a highly nervous temperament and there is any tendency toward undue irritability, it is well to give nerve sedatives : Tetanus, a frequent sequellæ to a punctured wound of the foot, is not thus caused solely on account of the situation of the injury. But the ratio of pedal traumations is greater than in any other part; or if not more frequent, it is seldom they are not made manifest; whereas in other parts we may not so readily appreciate them. Then again, their situation has added to their aptitude to contamination with such articles as may have these germs and this facility of introduction. This "concomitancy," or to be more correct "frequent sequence," has made wounds of the feet, as the cause of tetanus, difficult to acclude.

How often do we see wounds on any or all parts having no such sequence; but let there be a history of tetanus in the stable, and the inquiry ever so slight, then grave anticipations are generally realized. Sometimes even with such a history tetanus may not follow, and certain practitioners may, on the strength of this, argue against the germ theory being applied to tetanus. Such persons should realize that theirs was an exceptional case and that there are probably "immunes" from all of the "specific diseases." Thus idiopathic tetanus is obliterated, and what formerly was considered idiopathic is probably traumatic and is most assuredly, as is all tetanus, of bacterial origin. The most general treatment of tetanus now practiced is to place the animal in a darkened box-stall impervious to draughts and noises. I have found hydrocyanic acid hypodermically administered beneficial. If this rigidity can be diagnosed in its incipiency, and complete trismus has not set in, it is well to turn the animal to pasture and let him keep constantly moving his maxillaries. If in winter I would keep hay continually before them when in this condition. It is recommended to excise the cicatrix in tetanus. I myself have applied a strong blister to the healed-over wound with good results.

The paper was well received and a vote of thanks was tendered the essayist.

Dr. Bridge does not believe in great heroic cuttings, but only large enough to drain the pus and dress with phenol creosote. Drs. Hoskins and Sellers want free openings, with antiseptic dressings. The subject was discussed *pro* and *con* as regards the extent of the openings. On account of the lateness, Dr. Lintz' case to cite was laid over until the next meeting. Dr. Werntz will read at the January meeting.

